# Acute Compartment Syndrome Following Non-Displaced Proximal Ulnar and Distal Radial Fractures in a Four-Year-Old Girl

**DOI:** 10.14740/jmc5223

**Published:** 2025-12-24

**Authors:** Khalid Aloqeely, Amal Yousif, Fatima Aljaziri

**Affiliations:** a Department of General Pediatrics, King Saud University Medical City, Riyadh, Saudi Arabia; b Department of Emergency Medicine - Pediatric Emergency Unit, King Saud University Medical City, Riyadh, Saudi Arabia; c Pediatric Emergency Department, Maternity and Children Hospital, Dammam, Saudi Arabia

**Keywords:** Acute compartment syndrome, Non-displaced fractures, Early recognition, Intervention, Children

## Abstract

Acute compartment syndrome (ACS) is a rare but limb-threatening emergency in children, usually associated with displaced fractures, crush injuries, or high-energy trauma. Prompt recognition and fasciotomy are essential to prevent permanent disability. An unusual case of ACS after non-displaced fractures is presented, challenging traditional risk factors. A healthy 4-year-old girl presented 12 h after a 2-m fall with severe forearm pain, swelling, an absent radial pulse, delayed capillary refill (3 - 4 s), and cold digits. Radiographs showed non-displaced proximal ulna and distal radius fractures. Emergency fasciotomy was performed based on clinical findings of ACS. ACS can occur in children after non-displaced fractures, even without conventional risk factors. Clinicians should rely on careful neurovascular assessment and clinical suspicion rather than fracture type or mechanism alone. Early recognition and surgical intervention are critical to preserve limb function.

## Introduction

Acute compartment syndrome (ACS) is a medical emergency characterized by elevated interstitial pressure within a closed osteofascial compartment, leading to decreased tissue perfusion and ischemia [1]. The pathophysiological cascade typically begins with edema or bleeding in a fixed-volume compartment, raising venous and tissue pressure. When compartment pressure exceeds capillary perfusion pressure, muscle and nerve ischemia ensue [1]. Although this model presents a plausible, linear cascade of events, it is important to recognize that more complex mechanisms for this pathophysiology have also been described in the literature [2].

In the upper extremity, ACS involves several anatomical considerations. The forearm contains three main compartments: the volar compartment, the dorsal compartment, and the mobile wad (brachioradialis, extensor carpi radialis longus, and brevis), which primarily contains muscles involved in elbow flexion and wrist extension. The hand alone has ten distinct fascial compartments [1].

Fractures are the principal cause of ACS [3, 4], although iatrogenic causes have also been reported [5]. Atraumatic causes including hereditary angioedema, cellulitis, and osteomyelitis have also been reported [4, 6-9]. The incidence is fortunately rare, affecting 0.3% of pediatric patients after trauma [10]. The median age at presentation is 12 years, with girls accounting for 20% of cases compared to boys [11]. The upper limb is involved in 40% of cases compared to the lower limb [12], and the hand is affected in 35% of cases compared to the forearm [4].

Supracondylar fracture (SCF) is classically associated with pediatric ACS, most often involving the volar forearm compartment, but its incidence is still only 0.1-0.3% [13, 14]. Reported risk factors include male sex, high-energy trauma, and concomitant humeral and forearm fractures [15, 16].

Diagnosis of ACS is primarily clinical, based on findings such as severe pain, pain with passive movement, swelling, paresthesia, and diminished pulses [3]. Ancillary investigations may help when findings are equivocal [17]. Diagnosing ACS in children under 3 years is particularly challenging due to limited ability to verbalize symptoms [18]. Increased analgesic requirements and irritability have been reported as early indicators in this age group [19].

The prognosis of ACS depends on etiology and time to diagnosis [20]. Outcomes are generally favorable in pediatric cases, with one study reporting 74% excellent outcomes and 22% fair outcomes even when treatment was delayed [4]. No previous reports were identified describing ACS developing in a pediatric patient as a consequence of non-displaced forearm fractures [21, 22].

## Case Report

This case report was prepared in accordance with institutional ethical guidelines and the CARE checklist for case report standards.

A previously healthy 4-year-old girl presented to the emergency department with acute left forearm pain following trauma sustained 12 h earlier. On the evening before presentation, she jumped from a cabinet approximately 2 m in height and landed on a carpeted floor. She primarily struck her left elbow and wrist, experiencing immediate severe pain while retaining the ability to move her hand freely. No other injuries were sustained, and there were no signs of trauma to other body regions.

Due to logistical constraints, the family could not seek immediate medical evaluation. About 3 h post-injury, the patient was assessed at a nearby primary care center. Since radiological imaging was unavailable, her family returned home. Over the next several hours, the pain progressively worsened. Approximately 1 h later (4 h post-injury), her left hand became swollen, and she experienced significant sleep disturbance overnight.

The following morning, about 12 h post-injury, her mother brought her to the emergency department, where she was triaged as Canadian Triage and Acuity Scale (CTAS) 2.

On physical examination, the patient appeared in severe distress with pain unrelieved by oral analgesics given earlier. She was unable to follow commands due to intense pain, making examination difficult. Marked swelling was noted over the distal third of the left forearm, wrist, and hand, with erythema and discoloration around the wrist. The hand was cold, with diffuse severe tenderness. She attempted to move her digits, wrist, and elbow but with extreme difficulty due to pain and was unable to be consoled.

**Figure 1:**
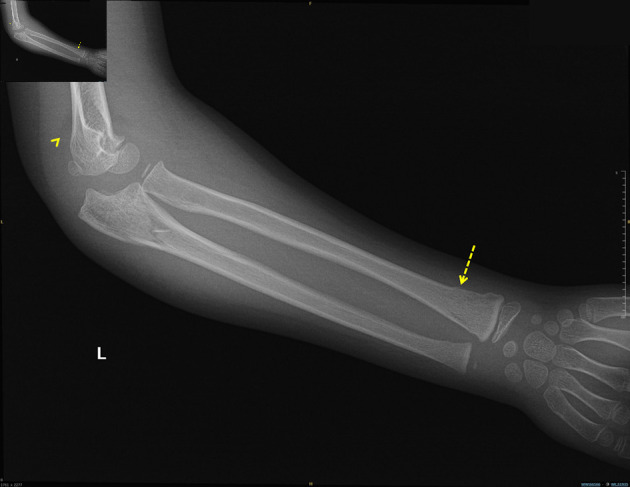
Lateral elbow radiograph demonstrating the posterior fat pad sign (arrowhead) and distal radial fracture (dashed arrow).

**Figure 2:**
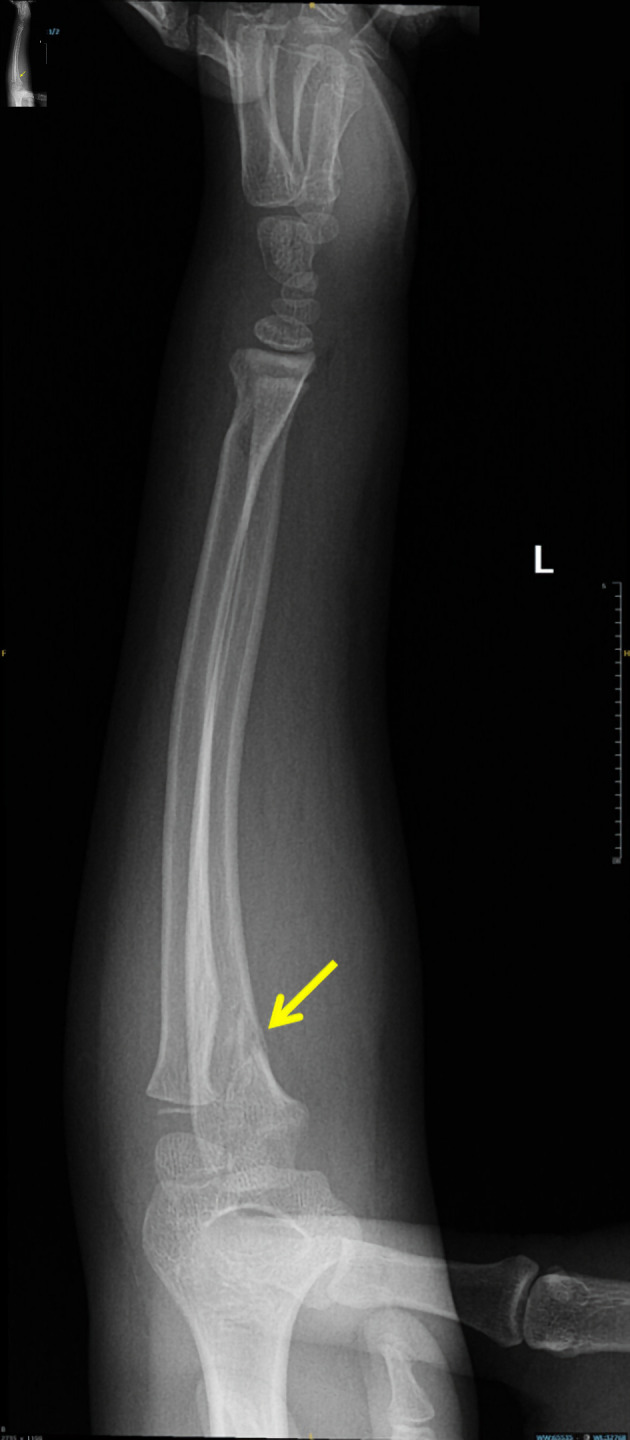
Anteroposterior forearm radiograph demonstrating proximal ulnar fracture (arrow).

Capillary refill was delayed (3 - 4 s), and the radial pulse was absent on palpation and undetectable by Doppler. However, pulse oximetry readings were obtainable from the left thumb, middle, and ring fingers. She was afebrile and hemodynamically stable except for tachycardia (heart rate 120 beats per minute (bpm)). Urgent radiographs demonstrated non-displaced proximal ulnar and distal radial fractures of the left forearm (Figs. 1, 2).

Preoperative laboratory studies were obtained immediately (complete blood count, coagulation profile, urea and electrolytes), and the patient received intravenous fluids and analgesia. Both the orthopedic and vascular surgery teams evaluated her immediately upon arrival, and she was transferred to the operating room for emergency fasciotomy. Multidisciplinary fasciotomies of the hand and forearm were performed under the care of orthopedic, vascular, and plastic surgery teams. Post-decompression assessment with Doppler confirmed intact ulnar and radial pulses.

Postoperatively, the patient required additional interventions, including serial surgical debridement and subsequent skin grafting. Despite these extensive efforts, she developed profound motor deficits in the affected hand, characterized by complete loss of active wrist and digit flexion and extension. The hand remained in a non-functional position with significant stiffness limiting passive range of motion. She was subsequently transferred to a specialized rehabilitation facility for intensive physiotherapy and ongoing care.

## Discussion

This patient presented with high-energy trauma to the left forearm, accompanied by classic clinical features of ACS (severe pain, swelling, and absent radial pulse) following a 12-h delay in presentation. Radiographs revealed two non-displaced fractures (proximal ulna and distal radius). No previous reports were identified describing ACS developing in a pediatric patient as a consequence of non-displaced forearm fractures.

A systematic literature review was conducted using PubMed, EMBASE, and Google Scholar from inception to December 2025 with search terms: “acute compartment syndrome,” “pediatric,” “non-displaced forearm fracture,” “radius,” and “ulna.” The search was limited to English-language publications. No prior pediatric cases of ACS from combined non-displaced proximal ulnar and distal radial fractures were identified.

A recent systematic review of combined humeral and forearm fractures confirmed that all reported cases involved displacement [21].

A plausible explanation for ACS in this case is the presence of multiple fractures within the same extremity. As previously described, bleeding and edema within a closed osteofascial compartment can elevate pressure above capillary perfusion pressure, ultimately causing ischemia [1]. Taken together, the high-energy mechanism, potential soft tissue damage, and multiple fractures within one limb likely contributed to the development of ACS in this child and highlighted the importance of obtaining a detailed history of the mechanism of injury.

This case underscores the principle that ACS is a clinical diagnosis. Radiographs and fracture morphology cannot reliably exclude ACS. Absence of classic risk factors should not delay surgical evaluation. Any delay in recognition or intervention can result in irreversible tissue injury and permanent disability, as reflected in the patient’s persistent motor deficits despite timely fasciotomy and multidisciplinary care.

While this single case report has inherent limitations, it demonstrates an important clinical principle: ACS risk assessment should prioritize mechanism of injury and clinical findings over radiographic fracture patterns, particularly in pediatric patients

### Conclusions

This case highlights the importance of careful neurovascular assessment in pediatric patients with fractures. ACS can develop even after non-displaced fractures and in the absence of recognized risk factors. Clinicians should maintain a high index of suspicion and prioritize clinical findings over imaging. Taken together, the high-energy mechanism, potential soft tissue damage, and multiple fractures within one limb likely contributed to the development of ACS in this child. These factors also highlight the importance of obtaining a detailed history of the mechanism of injury. Multiple non-displaced fractures in the same limb may precipitate ACS, as demonstrated in this report. Early recognition and prompt surgical intervention remain critical to preserving limb function.

## Data Availability

Any inquiries regarding supporting data availability of this study should be directed to the corresponding author.
